# Oral administration of berberine represses macrophage activation-associated benign prostatic hyperplasia: a pivotal involvement of the NF-κB

**DOI:** 10.18632/aging.203434

**Published:** 2021-08-19

**Authors:** Bo-Ram Jin, Hyo-Jin An

**Affiliations:** 1 Department of Pharmacology, College of Korean Medicine, Sangji University, Wonju-si, Gangwon-do 26339, Republic of Korea

**Keywords:** benign prostatic hyperplasia, berberine, inflammation, proliferation, macrophage

## Abstract

Benign prostatic hyperplasia (BPH) is one of the most common chronic diseases in men over the age of 50. Clinical studies have suggested that chronic inflammation is associated with BPH pathoprogression. Berberine (BB) is a natural compound found in *Berberis vulgaris*, *Coptis chinensis* and *Phellodendron amurense.* Although several studies have documented that BB may be effective for inflammation, the effects of the oral administration of BB on BPH are not fully understood. The effects of BB on chronic prostatic inflammation were evaluated in a testosterone-induced BPH animal model. Orally administered BB alleviated the pathological alterations induced by BPH and significantly suppressed the expression of inflammatory markers while enhancing the expression of antioxidant factors. Furthermore, BB regulated the activation of macrophages via NF-κB signaling pathway inhibition in the BPH rat model. The effects and underlying signaling pathway of BB in RWPE-1 cells exposed to macrophage conditioned medium (CM) were also demonstrated *in vitro*. While CM stimulation induced prostatic cell proliferation and upregulated the expression of inflammatory factors, BB exerted anti-proliferation and anti-inflammatory effects in RWPE-1 cells. These findings propose that BB suppresses androgen-dependent BPH development by targeting NF-κB-mediated pro-inflammatory signaling.

## INTRODUCTION

Benign prostate hyperplasia (BPH) is common condition among men above the age of 50. The cumulative incidence of BPH has been assumed to range from 50% in men aged 40–50 years up to 90% in men above the age of 80 [1]. It is recognized as mainly a proliferative disease, with prostate enlargement resulting in obstructive lower urinary tract symptoms and bladder outlet obstruction [2]. While prostatic hyperplasia seriously damages the quality of life, the pathogenesis of BPH remains unclear. It has commonly been assumed that androgen receptor (AR) signaling exerts a powerful effect upon the pathogenesis of BPH. Finasteride, a 5α-reductase inhibitor (5ARI), which represses transformation of testosterone to dihydrotestosterone (DHT), is used primarily in the treatment of BPH [3]. However, AR signaling pathway is not the sole regulator of prostatic growth, as evidenced by the fact that over 25% of patients do not respond to 5ARIs [4].

The etiology of BPH is linked to various factors, including age-dependent changes in hormone level, reorganization of tissue, and the involvement of various growth factors. Among them, chronic inflammation is considered a potential initiator of BPH pathoprogression. Although there is still no consensus as to whether inflammation is a parallel occurrence or an immediate cause, some research suggests that a significant link exists between inflammation and BPH. This is in accordance with several studies in which repeated tissue damage due to inflammatory processes was found to expedite compensatory cellular proliferative programs, resulting in hyperplasia [5, 6]. Inflammation is also continuously affected by androgens and other changes within metabolic conditions; however, the precise signaling pathways remain to be elucidated [7].

Histologically, BPH is considered a nodular transformation, with hyperplasia of both the stroma and epithelial components of the prostate [8]. Several studies have suggested a possible significance of immunocompetent cells, and the notion that both stromal and epithelial elements respond to inflammatory processes with nodular hyperplasia [9]. It has been demonstrated that the increased infiltration of macrophages mainly affects the propagation of BPH [10]. Macrophage activation in prostate tissue secretes a large number of inflammatory cytokines that crosstalk with prostate cells and induce the proliferation of prostatic cells [11, 12]. Meanwhile, studies are increasingly showing that macrophages and reactive oxygen species (ROS) exert a dual role in prostatic disease progression [13, 14]. ROS play a role in the regulation of M1 phenotype, while M2 macrophages relieve inflammation, which favors oxidative metabolism [15]. However, there is little published data on the role of macrophages and ROS in regulating the development and progression of BPH.

Berberine (BB) is an isoquinoline alkaloid that is predominantly found in esculent and medicinal plants, including *Phellodendron amurense*, *Coptis chinensis* and *Berberis aristate* [16]. Recently, *in vivo* and *in vitro* evidence has shown that BB has potent antioxidant, anti-inflammatory, and antitumor effects [17, 18]. Interestingly, some studies have found that the effects of BB are attributed to the suppression of the NF-κB pathway induced by various inflammatory agents and carcinogens [19, 20]. A previous study also showed that BB can suppress the ROS-dependent inflammatory response by inhibiting NF-κB signaling in macrophages [21, 22]. A recent study showed that BB inhibited the anti-apoptotic pathway involving the HO-1/NF-κB-mediated signaling pathway in prostate cancer cells [18]. Additionally, BB injected into the inguinal region was found to ameliorate testosterone-induced BPH via the modulation of androgen signaling, indicating that BB may have an effects on prostatic diseases [23]. However, studies investigating the precise molecular mechanism underlying the anti-inflammatory and anti-proliferative effects of oral administration of BB on BPH are lacking.

The current study explored the anti-inflammatory and anti-proliferative effects of BB on the pathoprogression of BPH using a testosterone-induced BPH rat model. Furthermore, to demonstrate the precise molecular mechanism of BB, we established a macrophage conditioned medium (CM)-treated prostatic cell model.

## MATERIALS AND METHODS

### Animals

Sprague-Dawley rats (aged 6 weeks; 200 ± 20 g) were obtained from Daehan Biolink Co. (Daejeon, Korea). The animals were randomly allocated to four per cage and housed under conditions that were in accordance with the guidelines for the care and use of laboratory animals. The animal study was designed and performed in accordance with the principles of the National Institutes of Health and with approval from the Institutional Animal Care and Use Committee (IACUC) of Sangji University (IACUC Animal approval protocol #2014-22). Rats were randomly assigned to five groups (eight mice per group) following a 1 week acclimatization period to the laboratory conditions. Con group were split and stitched up without detaching the testicles after anesthesia using zoletil 50; the rats in the other groups were castrated. After a 1 week recovery period, the rats in the BPH-induced groups were injected with testosterone propionate (10 mg/kg/day, s.c.) alone or along with BB or Fina for 4 weeks, except weekends. At the end of the experimental period, all animals were sacrificed after anesthetization with zoletil 50 (i.m. 20 mg/kg).

### Histological analysis

The pieces of prostate were used for hematoxylin and eosin (H&E) staining. The histological analysis was carried out as described previously [18].

### Serum levels of dihydrotestosterone analysis

The DHT levels in the serum were determined using ELISA kits (CUSABIO Life Science, Houston, TX, USA). The assays were performed following the manufacturer’s recommendations [24].

### RNA extraction and quantitative real-time PCR

Total RNAs were isolated from the prostatic tissues of rats and RWPE-1 prostatic cells. Complementary DNA was synthesized according to the manufacturer’s instructions. Specific rat and human primers were designed, which are shown in Table 1. Quantitative real-time polymerase chain reaction (qRT-PCR) was performed as previously described [25].

**Table 1:** Primer sequences.

**Gene name**	**Sense primers**	**Anti-sense primers**
**rats 5α-reductase 2**	GGCAGCTACCAACTGTGACC	CTCCCGACGACACACTCTCT
**rats NOS2**	CATTGGAAGTGAAGCGTTTCG	CAGCTGGGCTGTACAAACCTT
**rats COX2**	TGTATGCTACCATCTGGCTTCGG	GTTTGGAACAGTCGCTCGTCATC
**rats GAPDH**	TGA TTC TAC CCA CGG CAA GT	AGC ATC ACC CCA TTT GAT GT
**rats CD68**	ACAGACAGTGAAGGCTCAAAGATG	CCCACCGTGTTCGACAT
**rats IL-6**	TGAACAGCGATGATGCACTG	AGAAACGGAACTCCAGAAGACC
**rats IL-1β**	TGAACAGCGATGATGCACTG	AGAAACGGAACTCCAGAAGACC
**rats CD206**	AACGTTCGCTGATGCAAACC	AGGCCTCAATCCAACCAAAC
**rats ARG1**	AAAGGTCCCGCAGCATTAAG	TTGAAAGGGGCTGTCATTGG
**rats IL-10**	AAATTGAACCACCCGGCATC	CGAGGTTTTCCAAGGAGTTGC
**rats HO-1**	GCGAAACAAGCAGAACCCAG	GCCTCTGGCGAAGAAACTCT
**rats SOD-1**	TTTGCACCTTCGTTTCCTGC	TCCCAATCACACCACAAGCC
**rats NQO-1**	GGCTGGTTTGAGAGAGTGCT	TGAAAGCAAGCCAGGCAAAC
**rats GPx-1**	ACAGTATGTCTGCTGCTCGG	ATCGGGTTCGATGTCGATGG
**rats GAPDH**	TGATTCTACCCACGGCAAGT	AGCATCACCCCATTTGATGT
**human AR**	GAGCCAGGTGTAGTGTGTGC	TCGTCCACGTGTAAGTTGCG
**human PSA**	ATAGGATTGCCCAGGCAGAA	CTAAGGGTAAAAGCAGGGAGAGAGT
**human PCNA**	TTAAACGGTTGCAGGCGTAG	AGGAAAGTCTAGCTGGTTTCGG
**human Bcl-xL**	ATCCACTCTACCCTCCCACC	GGGAGTGAGGACTCTAGCCA
**human IL-6**	CCGGGAACGAAAGAGAAGCT	AGGCGCTTGTGGAGAAGGA
**human TNF-α**	GCTGGAGAAGGGTGACCGAC	GTTCGTCCTCCTCACAGGGC
**human IL-1β**	TGGACCTCTGCCCTCTGGAT	GGCAGGGAACCAGCATCTTC
**human NOS2**	GGTAGAGGCCTGGAAAACCC	AGCTCATCCCCTTCTCCCAT
**human COX2**	AATGGGGTGATGAGCAGTTG	TAGCCACTCAAGTGTTGCAC
**human β-actin**	GGCCAGGTCATCACCATTGG	CTTTGCGGATGTCCACGTCA

### Western blotting

Extracted protein lysates were separated by SDS-PAGE and transferred to PVDF membranes as described previously [26].

### Immunofluorescence (IF)

IF analysis was conducted using prostate paraffin sections and RWPE-1 cells sample as described previously [27].

### Cell viability assay

The viability of cells was determined using the colorimetric MTT assay according to the manufacturer’s instructions.

### Cell culture and CM culture

The normal human prostatic epithelial cell line RWPE-1 and THP-1 were cultured as described previously [23]. To make CM, after adding 100 ng/ml phorbol 12-myristate 13-acetate (PMA) into THP-1 cells for 48 h, and the medium of THP-1 cells was collected by centrifuging at 4000 rpm/min for 10 minutes. RWPE-1 cells were cultured with CM in the absence or presence of BB for the indicated times.

### Cell apoptosis assay

Apoptotic cells were measured using a Muse annexin V and Dead cell kit (Luminex, TX, USA) according to the manufacturer’s instructions.

### Luciferase assay

RWPE-1 cells were co-transfected with NF-κB-Luc reporter plasmid vector and the phRL-TK plasmid (Promega, Madison, WI, USA) using 4D-Nucleofector^™^ (Lonza, Basel, Switzerland) according to the manufacturer’s instructions. 16 h after transfection, the cells were treated with CM with or without BB for 30 min. Cells were rinsed with cold PBS, lysed, and the luciferase activity was determined with the Promega luciferase assay system (Promega) according to the manufacturer’s instructions.

### Statistical analyses

Experiments were performed in triplicate, and representative data are indicated as the means ± SD. Statistical significance analysis was determined using analysis of variance (ANOVA) and Dunnett’s post-hoc test. Data were taken to be significant where P < 0.05.

## RESULTS

### Oral administration of BB suppressed androgen-dependent prostatic enlargement in BPH rat model

**Figure 1:**
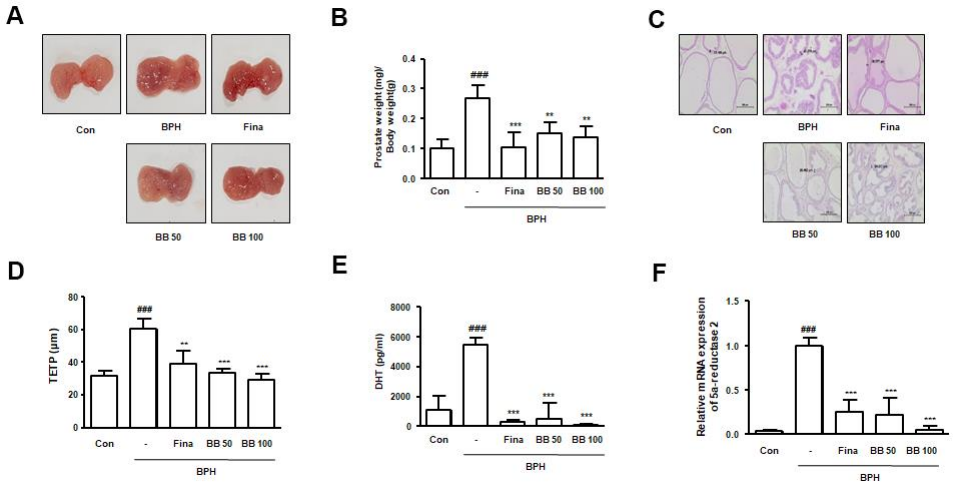
**Effects of BB on prostate gland enlargement via androgen-dependent manner in testosterone-induced BPH rat model.** The animals were randomly divided into five groups: Con group, BPH group (testosterone-induced BPH rats), Fina group (testosterone-induced BPH rats administered with Fina 5mg/kg), BB 50 group (testosterone-induced BPH rats administered BB 50 mg/kg), and BB 100 group (testosterone-induced BPH rats administered BB 100 mg/kg). (**A**) Representative photograph of prostate tissues from the testosterone-induced BPH rat model. (**B**) The prostate weight-to-body weight ratio was estimated. (**C**) Histopathological analysis was conducted using H&E. Original magnification 100×. (**D**) Based on histological analysis, the level of TETP was measured and represented. (**E**) Serum DHT levels were determined using an ELISA kit. (**F**) The mRNA level of 5α-reductase 2 was quantified using qRT-PCR analysis. ^###^ P < 0.001 versus the Con group; ** P < 0.01, *** P < 0.001 versus the BPH group.

To evaluate the therapeutic effects of BB, we set up a testosterone-induced BPH animal model with similar characteristic to human BPH. Rats were administrated with testosterone for 4 weeks, with or absent Fina and BB. It can be seen from the Figure 1A that the BPH group exhibited a notable expanded volume and hyperemia of the prostate contrasted to the Con group. Against BPH group, the groups orally administered Fina, BB 50, and BB 100 groups showed alleviated BPH-induced pathological signs. In the BPH group, a significant augmentation in the value of prostate weight (mg)/body weight (g) was also found as against the Con group (Figure 1B). However, the treatment of Fina, BB 50, and BB 100 notably reduced the index of prostate weight. As observed by H&E staining, rats from BPH group showed typical hyperplastic patterns, including multilayered epithelium and reduced glandular luminal area, and hyperplastic signs were descried in the Fina, BB 50, and BB 100 groups (Figure 1C). Consistently, TETP was significantly elevated in the BPH group, whereas a decrease was observed in the Fina, BB 50, and BB 100 groups (Figure 1D). The highest levels of DHT production and 5α-reductase 2 mRNA were observed in the BPH group compared with the Con group. In contrast, the treatment of Fina, BB 50, and BB 100 groups normalized these levels (Figure 1E, 1F).

### Oral administration of BB regulated prostate cellular proliferation in BPH rat model

**Figure 2:**
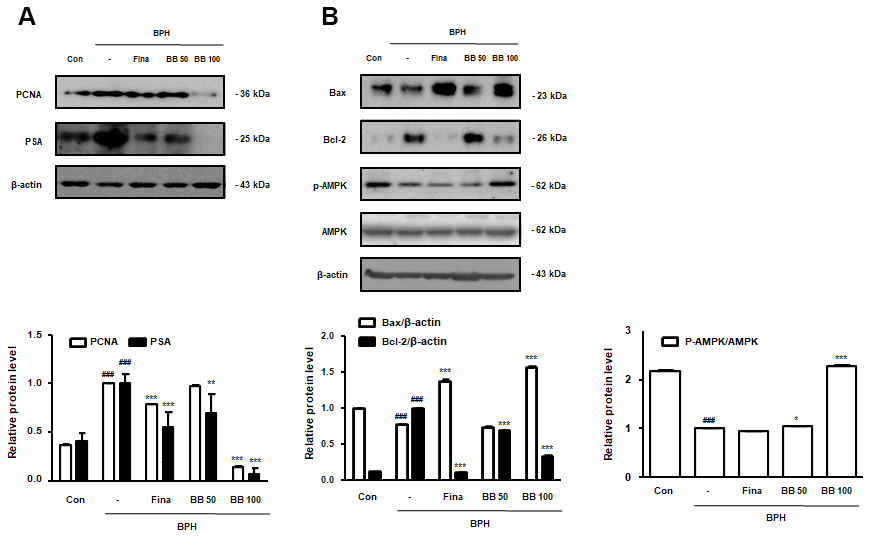
**Effect of BB on cell proliferation in testosterone-induced BPH rat model.** Western blot analysis of (**A**) PCNA, PSA (**B**) Bax, Bcl-2, p-AMPK, and AMPK. Data are expressed as means ± SD. ^###^ P < 0.001 versus Con group; * P < 0.05, ** P < 0.01, *** P < 0.001 versus the BPH group.

To find out the repressing effect of BB on cellular proliferation in prostate tissues from BPH rats, we estimated the protein expression of PCNA and PSA. Against Con group, the BPH group significantly augmented PCNA and PSA protein expression. However, the oral administration of Fina, BB 50, and BB 100 notably suppressed protein overexpression (Figure 2A). In agreement with our previous study, the BPH group exerted a significant imbalance of Bax to Bcl-2 in comparison to the Con group, whereas the treatment of Fina, BB 50, and BB 100 clearly restored this balance. In addition, the BPH group significantly decreased the phosphorylation of AMPK compared to the Con group. However, BB-treated groups showed restored AMPK phosphorylation compared with the BPH group (Figure 2B).

### Oral administration of BB inhibited overexpression of NF-κB-mediated pro-inflammatory markers in BPH rat model

**Figure 3:**
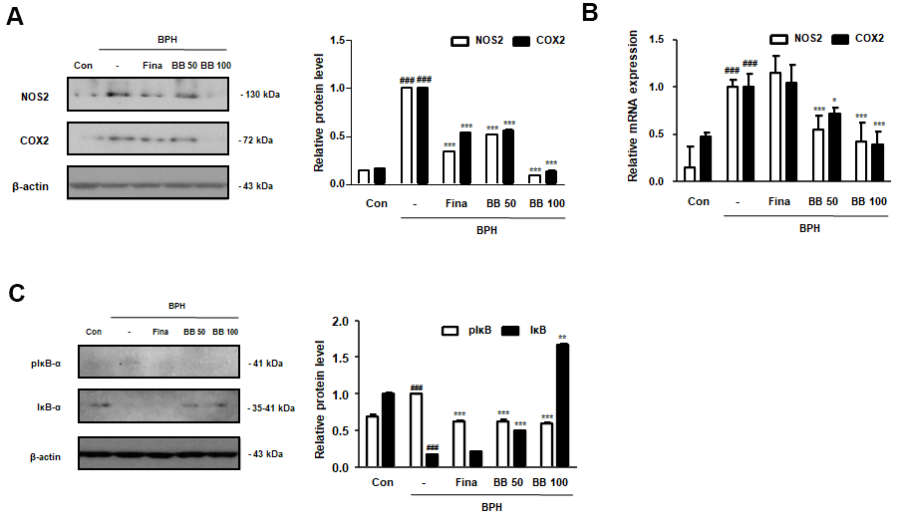
**Effect of BB on the expression of NF-κB-mediated inflammatory markers in testosterone-induced BPH rat model.** (**A**) Immunoblotting results showing the protein levels of NOS2 and COX2 in prostatic tissues. Densitometric protein levels are represented as means ± SD, and the plots of each protein are shown. (**B**) The NOS2 and COX2 mRNA levels in the prostate were quantified by qRT-PCR analysis. Values are represented as means ± SD of data from three separate experiments. (**C**) Western blot analysis of pIκB-α and IκB-α in prostatic tissue. ^###^ P < 0.001 versus Con group; * P < 0.05, *** P < 0.001 versus the BPH group by ANOVA and Dunnett’s post-hoc test.

Prostatic inflammation is considered a principal factor in the development and progression of BPH [6]. As shown in Figure 3A, 3B, in the BPH group, the level of the principal inflammation factors, iNOS and COX-2 elevated compared to in the Con group. However, BB 50 and BB 100 exerted a dose-dependent inhibitory effect compared to the BPH group. Next, we investigated the repressive effect of BB on the NF-κB-mediated signaling. As demonstrated in Figure 3C, the induction of BPH led to the phosphorylation and degradation of free IκB proteins. As compared to BPH group, the activation of NF-κB signaling was suppressed following the oral administration of BB.

### Oral administration of BB regulated ROS-mediated macrophage activation in BPH rat model

**Figure 4:**
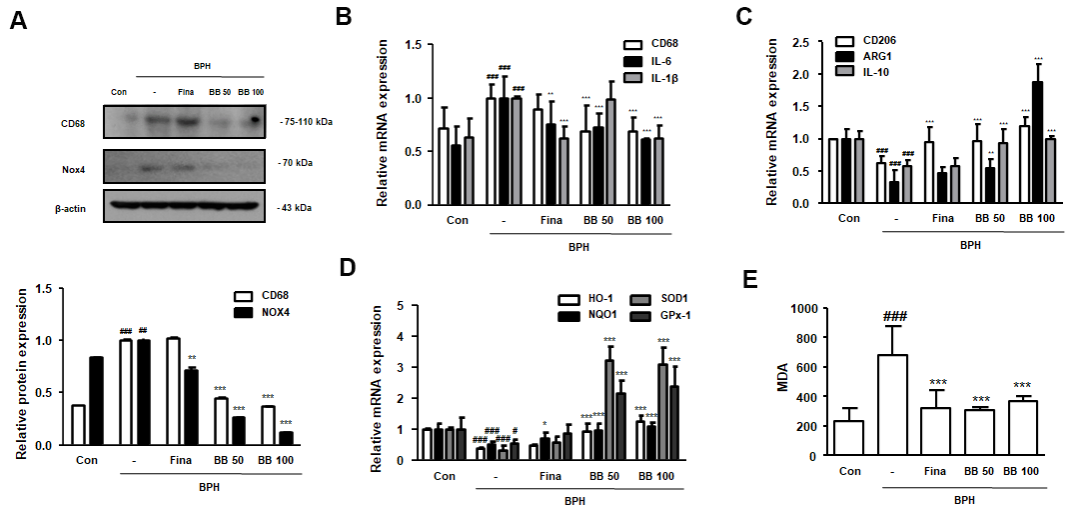
**Effect of BB on ROS-mediated macrophage activation in testosterone-induced BPH rat model.** (**A**) Western blot analysis of CD68 and NOX4. Data were represented as means ± SD. The mRNA levels of (**B**) CD68, IL-6 and IL-1β, and (**C**) CD206, ARG1 and IL-10 were quantified using qRT-PCR. (**D**) HO-1, NQO1, SOD1 and GPx-1 in prostatic tissues were determined using qRT-PCR analysis. β-actin was used as the housekeeping gene to standardize the gene expression levels. (**E**) MDA serum concentrations were analyzed using ELISA kit. ^#^ P < 0.05, ^##^ P < 0.01, ^###^ P < 0.001 versus Con group; * P < 0.05, ** P < 0.01, *** P < 0.001 versus the BPH group by ANOVA and Dunnett’s post-hoc test.

Further studies were conducted to demonstrate the molecular mechanism underlying inflammatory responses and apoptosis in BPH. In the BPH group, the protein expression of CD68 and NOX4 was significantly increased compared to in the Con group. In contrast, BB administration dose-dependently suppressed the protein expression of CD68 and NOX4, and the administration of Fina inhibited NOX4 protein expression only, but not CD68 (Figure 4A). Next, we analyzed the effect of BB on the expression of M1 and M2 macrophage subsets. As shown in Figure 4B, 4C, the mRNA levels of M1 macrophage markers CD68, IL-6, and IL-1β were higher in the BPH group than in the Con group, while the mRNA levels of M2 macrophage markers CD206, ARG1, and IL-10 were lower in the BPH group. However, the administration of BB significantly normalized these levels. We also found that the antioxidant genes HO-1, NQO, SOD1, and GPx-1 were significantly impaired by BPH induction, whereas BB administration significantly increased the expression of these antioxidant biomarkers (Figure 4D). Correspondingly, MDA, an indicator of oxidative damage, was increased in the BPH group as compared to its level in the Con group, while treatment of Fina, BB 50, and BB 100 significantly reduced the production of MDA (Figure 4E).

### Oral administration of BB suppressed the NF-κB activation within macrophage activation in BPH rat model

**Figure 5:**
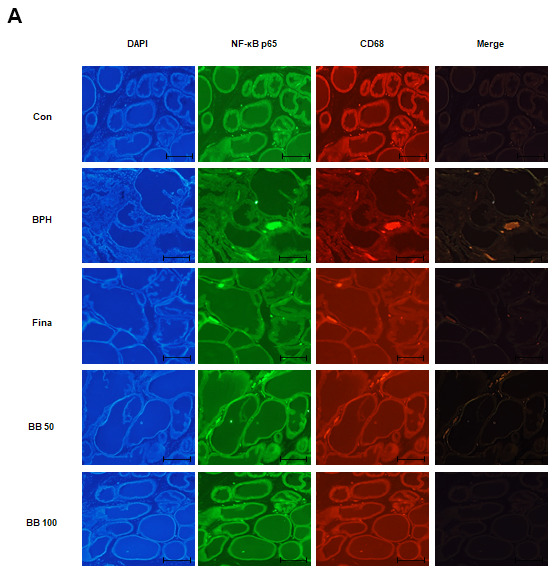
**Effect of BB on expression patterns of NF-κB p65 and CD68 in testosterone-induced BPH rat model.** (**A**) Fluorescent intensity and co-localization of NF-κB p65 and CD68^+^ cells in prostate sections from BPH rats. Whole-mount immunohistochemistry of the nucleus (blue), NF-κB p65 (green), and CD68 (red). The overlays show the extent of co-localization (yellow) of NF-κB p65 with macrophage marker CD68. Original magnification 100×.

NF-κB, a sensor of oxidative stress, induces macrophage activation under inflammatory processes. We investigated whether BB could inhibit the involvement of NF-κB within the macrophage activation status in the prostate of testosterone-induced BPH rats. The fluorescent intensity and co-localization of NF-κB p65 and macrophages (CD68+) were identified in prostatic tissues obtained from BPH rats. As can be seen from the Figure 5A, in the glandular luminal area, the BPH group overexpressed and co-localized NF-κB p65 and CD68 compared to the Con group. In contrast, Fina, BB 50, and BB 100 clearly suppressed the overexpression and co-localization of NF-κB p65 and CD68. These results highlight that the repressive effect of BB 100 on BPH via NF-κB activation within macrophage activation was superior to that demonstrated by the Fina group.

### Treatment of BB inhibited the cellular proliferation in THP-1-conditioned media treated prostate epithelial cells

**Figure 6:**
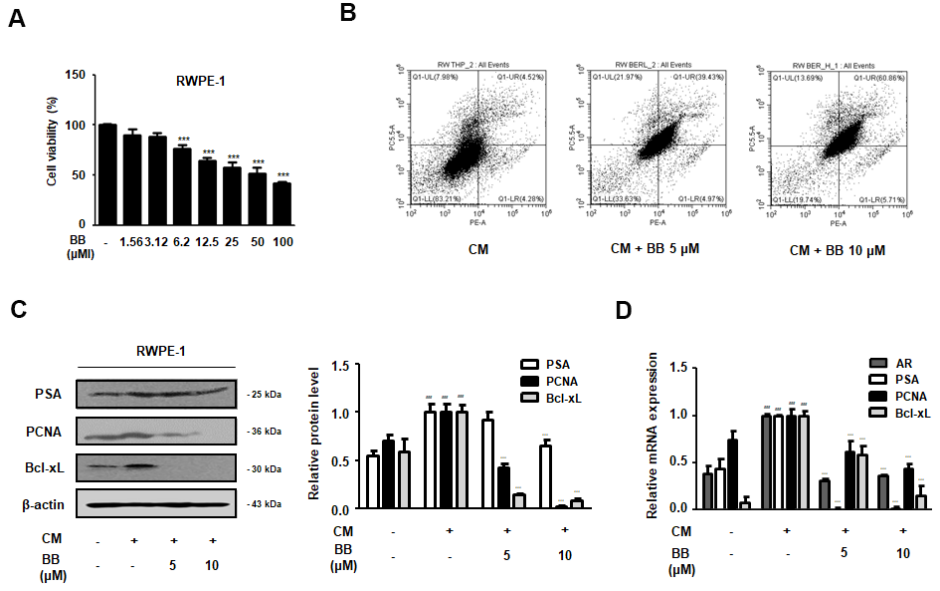
**Effect of BB on the apoptosis of prostatic cells in the CM-treated RWPE-1 cell model.** (**A**) Cell viability using MTT assay; n=6 per treatment across three separate experiments. (**B**) Apoptotic effect of BC inflammatory environments. The cells were stimulated with CM, with or without 5 and 10 μM BB for 24 h. (**C**, **D**) RWPE-1 were treated with CM for 3days, with or without BB (5, 10 μM). (**C**) Western blot analysis of PSA, PCNA, Bcl-xL. (**D**) qRT-PCR analysis of AR, PSA, PCNA, Bcl-xL. Data were represented as means ± SD. ^###^ P < 0.001 versus vehicle group; *** P < 0.001 versus the CM-treated group.

To investigate the repressive effect of BB on prostatic cell proliferation in inflammatory microenvironments, we set up an *in vitro* BPH model by stimulating RWPE-1 cells with THP-1 conditioned media. First, an MTT assay was used to confirm the effective dose of BB (1.56-100 μM) that abrogated the proliferation of BPH-1 cells (Figure 6A). As shown in Figure 6B, in contrast to the CM-treated group, BB 5 and 10 μM treatment notably increased the number of cells undergoing apoptosis by 39.42% and 60.86%, respectively. Next, we investigated whether treatment with BB could inhibit the BPH-relative gene expression in inflammatory microenvironments. The CM-treated group showed significantly increased protein expression of PSA, PCNA, and Bcl-xL, whereas treatment with BB dose-dependently suppressed their overexpression (Figure 6C). In addition, AR, PSA, PCNA, and Bcl-xL mRNA expression in the prostatic cells was significantly increased in the CM-treated group, whereas Fina treatment of BB markedly inhibited the mRNA expression of these genes (Figure 6D).

### Treatment of BB suppressed the NF-κB activation in CM-treated prostatic epithelial RWPE-1 cells

**Figure 7:**
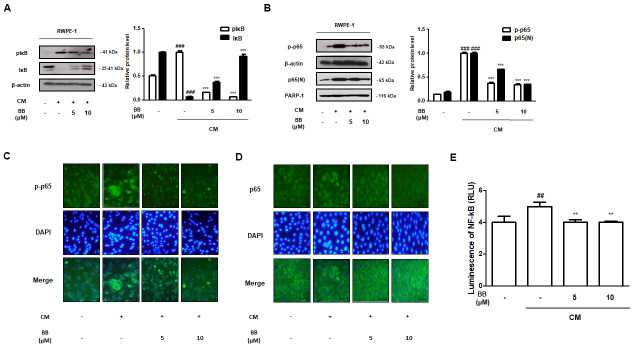
**Effect of BB on NF-κB signaling pathway in CM-treated RWPE-1 cell model.** (**A**, **B**) Prostatic cells stimulated with CM, with or absent 5 and 10 μM BB for 3 days. Western blot analysis of (**A**) pIκB, IκB and (**B**) p-p65, p65 in RWPE-1 cell total and nucleus lysates. ^###^ P < 0.001 as compared with the vehicle; *** P < 0.001 as compared with the CM-treated group using ANOVA and Dunnett’s post-hoc test. (**C**, **D**) IF staining of (**C**) p-p65 and (**D**) p65 in CM-treated RWPE-1 cells. (**E**) The luciferase activity levels were determined in pNF-κB-luc reporter-transfected RWPE-1 cells.

The animal study results revealed the potent therapeutic effects of BB, which inhibited the inflammatory responses by suppressing NF-κB activation in the prostatic tissues of BPH rats. To identify the precise molecular effect of BB on the NF-κB-mediated signaling *in vitro*, we exposed RWPE-1 cells to inflammatory conditions using THP-1 supernatant-derived CM. CM stimulation elevated the phosphorylation of IκB and p65, and induced the nuclear translocation of p65 in RWPE-1 cells, whereas treatment with 5 and 10 μM BB significantly repressed (Figure 7A, 7B). Consistently, CM-treated RWPE-1 cells strongly expressed fluorescent p-p65 and nuclear p65, but BB treatment notably suppressed p-p65 and p65 fluorescence intensity (Figure 7C, 7D). In addition, BB treatment was found to significantly inhibit NF-κB transcriptional activity in CM-treated RWPE-1 cells (Figure 7E).

### Treatment of BB inhibited inflammatory responses in CM-treated RWPE-1 cells

**Figure 8:**
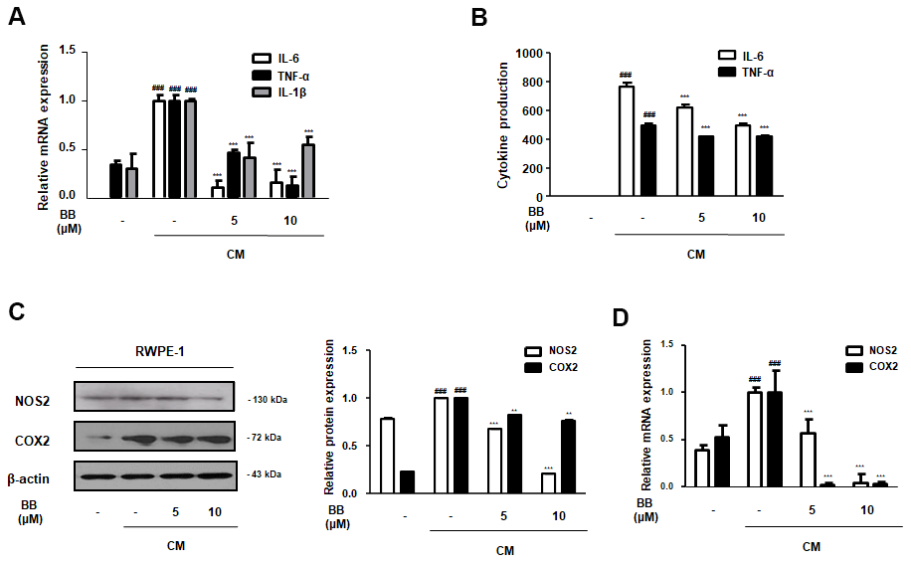
**Effect of BB on pro-inflammatory factors in CM-treated RWPE-1 cell model.** The prostatic cells were stimulated with CM, with or without 5 or 10 μM BB for 3 days. (**A**) The mRNA level of IL-6, TNF-α, and IL-1β in CM-treated RWPE-1 cells were analyzed by qRT-PCR analysis. (**B**) The released pro-inflammatory cytokines IL-6 and TNF-α were quantified using an ELISA kit in the supernatant from CM-treated RWPE-1 cells. The expression of (**C**) protein and (**D**) mRNA of NOS2 and COX2 were determined in CM-treated RWPE-1 cells. Each gene level was normalized to β-actin. Results are represented as the mean ± SD. ^###^ P < 0.001 versus vehicle group; ** P < 0.01, *** P < 0.001 versus the CM-treated RWPE-1 cell group.

Pro-inflammatory cytokines, such as TNF-α and interleukins, are under the control of transcriptional NF-κB. However, these cytokines also directly stimulate the activation of NF-κB signaling, and vice versa [28]. As shown in Figure 8A, prostatic cells under the inflammatory microenvironment overexpressed IL-6, TNF-α, and IL-1β mRNA, in contrast to normal conditions. However, treatment with 5 and 10 μM BB significantly abrogated these pro-inflammatory cytokines at the mRNA level. Additionally, a similar trend was observed in the supernatants. CM-treated RWPE-1 cells released excess production of IL-6 and TNF-α in culture supernatants, whereas BB 5 and 10 μM significantly suppressed the production of these cytokines (Figure 8B). As shown in Figure 8C, 8D, CM stimulation also promoted the upregulation of NOS2 and COX2 gene levels in RWPE-1 cells. In contrast, treatment with BB significantly and dose-dependently inhibited these CM-treated abnormal effects.

## DISCUSSION

This study explored the remedial effects of BB on BPH and its underlying molecular mechanisms in hyperplastic prostatic cells under the inflammatory microenvironment. To evaluate the effects of BB on the progression of BPH, we used a testosterone-induced BPH rat model and established a macrophage CM-treated prostatic epithelial cell model. We found that the oral administration of BB repressed the thickening of the prostatic epithelium, as well as the overproduction of DHT and the upregulation of 5α-reductase 2 mRNA levels, resulting in the suppression of pathological prostate enlargement in the BPH rat model. In addition, the oral administration of BB regulated proliferation and inflammation-related genes in these rats. Furthermore, ROS-mediated macrophage activation was found to be suppressed by the administration of BB via the NF-κB signaling pathway. *In vitro*, treatment with BB exerted anti-proliferative and anti-inflammatory effects by suppressing NF-κB signaling in macrophage CM-treated prostatic epithelial cells. These results provide evidence of the potential effects of BB as a novel naturally-derived product for the treatment of BPH.

Usually, prostatic enlargement in therapeutics for BPH is controlled through steroid management by blocking androgen signaling. Finasteride, a 5α-reductase inhibitor approved by FDA, has proven powerful in treating BPH, decreasing prostatic DHT levels, and reducing prostatic size [29]. Finasteride has also been used for the treatment of chronic prostatitis with prostatic enlargement [30, 31]. However, the use of this drug has been limited due to certain adverse effects, such as male infertility, impotence, and loss of libido [32]. It has been suggested that even finasteride may increase the risk of subsequent prostate cancer progression [33].

The upregulation of AR signaling can contribute to the pathogenesis of BPH. Therefore, the inhibition of androgen signaling by BB may promote the susceptibility of prostatic cells to current therapy. Recently, researchers have found that the treatment of the inguinal region with BB improves BPH by suppressing 5α-reductase in BPH-induced rats [23]. Meanwhile, previous studies have demonstrated that, in contrast to its significant pharmacological effects, the plasma concentration of BB is very low; on the other hand, the organ concentration of BB, as well as its metabolites, is higher than its level in the blood after oral administration, indicating that the efficacy of BB is potentially increased by oral administration [34]. BB is most commonly used for the treatment of clinical disorders, including diabetes, cancers, neurodegenerative diseases, and other diseases [35]. However, the pharmacological effects of the oral administration of BB on BPH remain poorly understood. For this reason, and after review the literature, we considered that it is necessary to demonstrate the effect and regulatory mechanism(s) of BB on BPH after oral administration.

In the present study, increases in the degree of proliferation and the upregulation of inflammatory-related factors were observed simultaneously, and affected one another in testosterone-induced BPH rats. The infiltration of CD68, a well-known macrophage-specific marker, has been reported in prostate tissues with glandular hyperplasia [36]. NOX4 was described as a novel inducible source of ROS in macrophages, and previous studies have reported that NOX4-derived ROS promoted pathogenesis of BPH [37]. Our results suggest that increased levels of CD68 and NOX4 due to excess androgen may enhance the proliferation of prostatic cells in the BPH rat model. In contrast, these results demonstrate that the activation of macrophages and inflammatory conditions were significantly suppressed by the oral administration of BB. Concurrently, antioxidant markers were restored in prostatic tissues with BPH by orally administering BB, thereby demonstrating the antioxidant effects of BB on the development of BPH.

Meanwhile, there are potential limitations that should be considered. Because it is difficult to investigate which inflammatory element is directly involved in prostatic hyperplasia, the observed results reflect the incorporated effects of macrophages and other alterations on androgen signaling in BPH. However, our hypothesis is still firmly supported by the relationship between inflammation and proliferation. To our knowledge, this study provides the first comprehensive assessment of the effects of the oral administration of BB on the pathoprogression of BPH. In addition, we found that macrophage-induced prostatic cell proliferation was counteracted by treatment with BB, suggesting that BB exerts the regulatory effects on prostatic cells under the inflammatory microenvironment.

## CONCLUSIONS

Our results provide evidence for the treatment of BPH using anti-inflammatory strategies, especially via the inhibition of the NF-κB signaling pathway. In addition, our finding indicated that the anti-BPH effect of BB is superior to that of finasteride, suggesting the possibilities of BB as a remedial agent for the treatment of BPH.
